# Surgical Management of Iatrogenic Pigment Dispersion Glaucoma

**DOI:** 10.5005/jp-journals-10008-1180

**Published:** 2015-01-15

**Authors:** Camille Van Mierlo, Luis Abegão Pinto, Ingeborg Stalmans

**Affiliations:** Resident, Department of Ophthalmology, University Hospitals of Leuven Leuven, Belgium; Assistant Professor, Department of Ophthalmology, Faculty of Medicine, Lisbon University, Lisbon, Portugal; Professor, Department of Ophthalmology, University Hospitals of Leuven Leuven, Belgium

**Keywords:** Pigment dispersion glaucoma, Iris chafing, IOL-exchange.

## Abstract

**Introduction:** Iatrogenic pigment dispersion syndrome generally originates from a repetitive, mechanical trauma to the pigmented posterior epithelium of the iris. This trauma can arise after intraocular surgery, most commonly due to an abnormal contact between the intraocular lens (IOL) and the iris. Whether surgical removal of this primary insult can lead to a successful intraocular pressure (IOP) control remains unclear.

**Methods:** Case-series. Patients with IOP elevation and clinical signs of pigment dispersion were screened for a diagnosis of iatrogenic IOL-related pigment dispersion.

**Results:** Three patients in which the IOL or the IOL-bag complex caused a pigment dispersion through a repetitive iris chafing were selected. In two cases, replacement of a sulcus-based single-piece IOL (patient 1) or a sub-luxated in-the-bag IOL (patient 2) by an anterior-chamber (AC) iris-fixed IOL led to a sustained decrease in IOP. In the third case, extensive iris atrophy and poor anatomical AC parameters for IOL implantation precluded further surgical intervention.

**Conclusion:** IOL-exchange appears to be a useful tool in the management of iatrogenic pigment dispersion glaucoma due to inappropriate IOL implantation. This cause-oriented approach seems to be effective in controlling IOP, but should be offered only if safety criteria are met.

**How to cite this article:** Van Mierlo C, Abegao Pinto L, Stalmans I. Surgical Management of Iatrogenic Pigment Dispersion Glaucoma. J Curr Glaucoma Pract 2015;9(1):28-32.

## INTRODUCTION

Pigment dispersion syndrome (PDS) is a condition where pigment is dispersed into the anterior chamber, eventually clogging the trabecular meshwork (TM). This continuous obstruction can ultimately increase intraocular pressure (IOP) and lead to a secondary open-angle glaucoma named pigment dispersion glaucoma (PDG).

The most common form of pigment dispersion is mainly due to a posterior bowing of the midperipheral iris leading to irido-zonular contact and subsequent pigment release. This process occurs mainly in young myopic white males, with identifiable clinical signs of this pigment release (iris transluminance, krukenberg spindle and homogenous TM pigmentation on gonio-scopy). However, this pigment dispersion can also result from intraocular surgery, in particular when there is an (inappropriate) implantation of an intraocular lens (IOL).^1,2^ In this case, the trauma to the iris posterior pigment epithelium is caused by this surgical implant. Accordingly, management is often directed at surgically removing this abnormal mechanical contact.

We report three cases of pigment dispersion originating from IOL-iris chafing. We provide details as to the diagnostic work-up to this unusual condition as well as to its surgical management, its outcomes and caveats.

## CASE REPORTS

### Case 1

A 58-year-old caucasian man was referred to our hospital because of intermittent episodes of blurred vision, halos and tearing associated with IOP elevation spikes (up to 30 mm Hg) and anterior chamber inflammation in the right eye [oculus dexter, (OD)]. Symptoms started 18 months before, after undergoing unilateral phacoemulsi-fication and IOL implantation (AcrySof SN6CWS, +18D). There was no relevant ocular or family history. At the time of referral, visual field testing was normal bilaterally and corrected visual acuity was 0.9 (snellen) on both eyes.

**Figs 1A to D:**
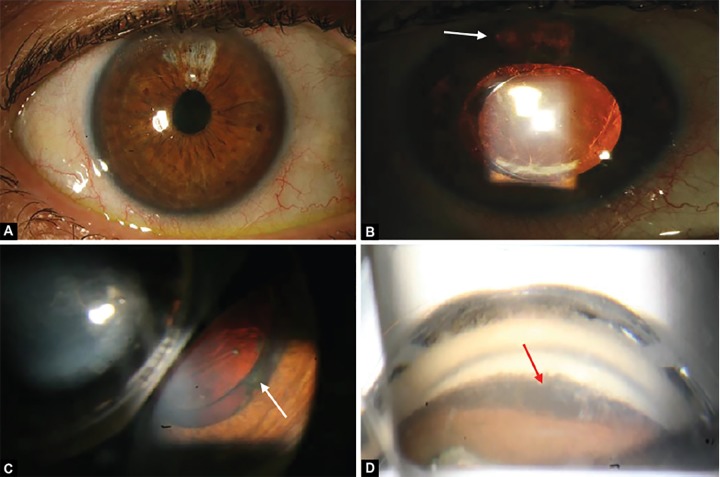
Anterior segment (A and B): (A) marked iris atrophy superior, (B) peripheral iris transillumination defects superiorly (arrow), matching the location of the haptic of the sulcus placed IOL. Image taken after pharmacological dilatation; Gonioscopy (C and D): (C) positioning of the IOL in the sulcus (arrow), (D) an open angle with homogenous heavy pigmentation of the trabecular meshwork (arrow)

Biomicroscopic examination revealed a unilateral OD Krukenberg spindle, with peripheral slit-like iris transillumination defects (with a prominent defect superiorly) and a positive Tyndall sign. Gonioscopy OD showed an open angle with 360° of homogenous hyperpigmentation of the TM, especially inferiorly (Figs 1A to D). A posterior capsu-lar tear inferonasal became apparent after dilation, as well as the positioning of the IOL in the sulcus. IOP OD was 44 mm Hg, without topical lowering therapy. No abnormal pigmentation was present on the otherwise normal, phakic OS, which presented with an IOP of 10 mm Hg.

The diagnosis of secondary pigment dispersion glaucoma was made, due to an extensive iris-IOL contact. Escalating medical therapy was unable to successfully control IOP, which led to the decision of surgically addressing the iatrogenic iris-IOL contact. An IOL exchange with implantation of an Artisan Irisclaw (+15D) in the anterior chamber was performed. Six weeks postoperatively, IOP had dropped to 10 mm Hg under triple topical therapy (brimonidine 0.2%-timolol 0.5% fixed combination, bimatoprost 0.3%), with no signs of anterior chamber inflammation.

### Case 2

A 47-year-old male with a history of a bilateral retinal detachment for which the patient underwent a bilateral vitrectomy with silicone oil tamponade, cataract surgery and silicone oil extraction between 2004 and 2006. He developed a bilateral steroid-dependent macular edema in 2013 and a sustained and uncontrollable IOP elevation (with spikes exceeding 30 mm Hg) despite maximal topical therapy, for which he was referred to our department. Surgery was scheduled for the eye with more elevated IOP (OD).

**Figs 2A to D:**
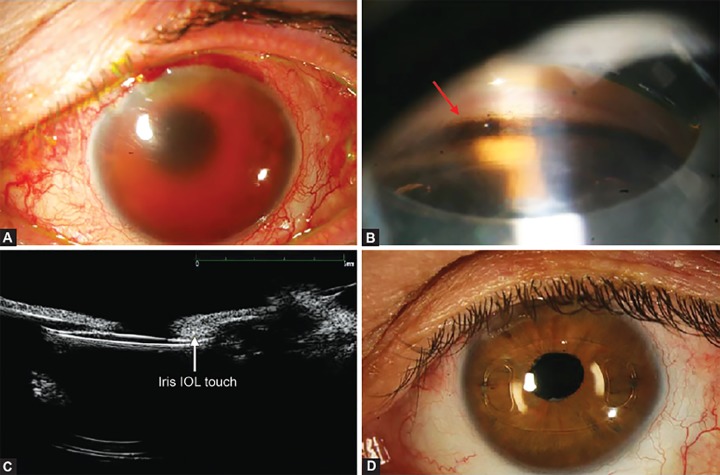
(A) Preoperative anterior segment photograph showing a diffuse anterior chamber hemorrhage, (B) gonioscopy demonst rating an open angle with inhomogenous heavy pigmentation of the trabecular meshwork (arrow), (C) preoperative ultrasound biomicroscopy showing contact between the anterior edge of the IOL optic and the posterior surface of the iris (arrow) and (D) postoperative (4 months) anterior segment photography of the Artisan IOL, without relapse of anterior chamber hemorrhage

Baerveldt tube implantation OD was uneventful. However, when planning surgery for the other eye, a mild IOL-subluxation was detected. A medical background check with the referring ophthalmologist revealed that the patient suffered from recurrent episodes of intraocular hemorrhages and anterior chamber inflammation since a blunt trauma to the eye in 2008. The multiple intraocular surgeries made iris atrophy interpretation difficult, with gonioscopy revealing mild synechiae and inhomogenous pigmentation. UBM examination revealed an IOL in the sulcus with a prominent IOL-iris contact. As in case 1, an IOL exchange was performed, with placement of an iris-claw Artisan lens (+14D) (Fig. 2). At 4 months after IOL exchange, pressures were 15 mm Hg under bitherapy (brinzolamide 1%-timolol 0.5% fixed combination), with no episodes of ocular inflammation during this follow-up. Accordingly, no filtering surgery was deemed necessary.

### Case 3

**Figs 3A to G:**
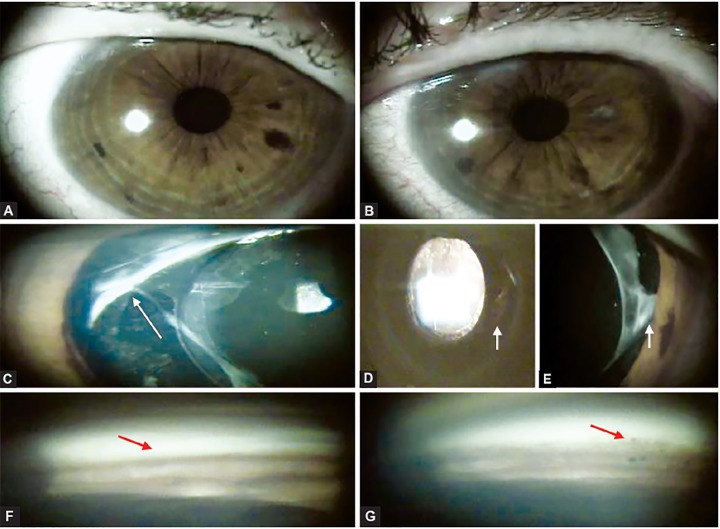
Anterior segment photo with no abnormal features visible in either eye (A, B), (C) right eye―haptic seen anteriorly to the lens capsule (arrow), (D and E) left eye―iris transillumination temporal inferior matching the location of the sulcus-placed IOL (arrows), gonioscopy (F and G) showing a heavy trabecular meshwork pigmentation on both eyes (arrows)

A 78-year-old woman was referred to our hospital because of recent glaucoma diagnosis 3 months before. As medical history, she had a bilaterally phacoemulsifi-cation surgery in 2008. At observation, pressures were above target bilaterally (OD 18 mm Hg and OS 26 mm Hg, both under triple therapy―dorzolamide 2% and timolol 0.5% fixed combination, and brimonidine 0.2%). At slit-lamp observation, an extensive oval peripheral iris atrophy was seen on both eyes. After dilation, these were matched to the underlying haptics of a single-piece IOL placed in the sulcus in front of a ruptured capsular bag. Gonioscopically, an extensive bilateral trabecular pigmentation was observed (Fig. 3). The workup for an IOL exchange revealed a bilateral decrease in endothelial cells (900-1100 cell/mm^2^). Combined with the iris atrophy, these parameters would significantly decrease the safety of the intended intraocular surgery due to potential cor-neal damage. Optimizing medical therapy (bimatoprost 0.1%% and brimonidine 0.2%-timolol, 0.5% fixed combination), including improved compliance, decreased IOP below the 18 mm Hg range (OD 16 mm Hg and OS 17 mm Hg). After properly discussing the pros and cons of a surgical intervention, the patient and physician decided for a ‘watchfully wait’ approach, postponing surgical intervention.

## DISCUSSION

Intraocular pigment dispersion has been widely known as a mechanism behind a sustained elevated IOP. While the mechanic trauma over the pigment-rich uveal/choroidal tissue causing such pigment release is mostly primary, it can also be caused by a disruption of the ocular anatomy during surgery. One of the surgeries more likely to cause such trauma to the posterior-chamber lining pigmented-tissues is cataract surgery, especially if a single-pieced IOL has been placed in the sulcus.

This phenomenon is scarcely described in the literature, with small, retrospective studies suggesting this pigment-release to occur with mostly abnormal IOL-bag complex position. In fact, while placement of an IOL in the sulcus has been consistently associated as a risk factor for this iris chafing, even a correct placement of the IOL cannot rule out this possibility, as secondary displacement of a haptic into the sulcus has been known to occur.^3,4^

In the past decade, several cases of PDS with IOP elevation have been reported after implantation of a single-piece hydrophobic AcrySof IOL in the ciliary sulcus.^5-8^ Several reasons have been advocated why placement of a single-piece acrylic hydrophobic AcrySof in the sulcus is contraindicated. Firstly, the haptics are large and thick, which facilitates contact with the posterior iris. Secondly, the haptic design is planar (instead of angulated posteriorly). Thirdly, the edges are sharp and the surface is unpolished. These factors increase the risk of mechanical trauma to the pigment epithelium and vasculature of the posterior iris and to the ciliary sulcus uveal tissue, leading to pigment dispersion, uveal inflammation and recurrent microhyphema’s and vitreous hemorrhages (uveitis-glaucoma-hyphema or UGH syndrome).^5,7-9^ Finally, the loop-to-loop diameter is often too small (13 mm) for a sulcus implantation, which allows dislocation.^5^ Conversely, when placed in the capsular bag, the edges are usually covered by the anterior and posterior capsules (if the capsulorhexis is centrally located and smaller than the size of the optic) and there is adequate support.^5-7^

Diagnosing the condition can be difficult. There are, however, several aspects that should alert the clinician for this possibility. First, the combination of an IOP elevation and abnormal pigment dispersion in an eye which had a cataract surgery. This is most striking when addressing a patient who is unilaterally pseudophakic. In such cases, the lack of pigment dispersion signs in the contralateral eye is highly suggestive of an iatrogenic PDS. The morphology of the iris transillumination could also suggest an iris chafing by the underlying haptic. Furthermore, the presence of an anterior chamber inflammation, often associated with iritis, is not normally seen in the primary form of the disease. Ultimately, a correct characterization of the interface between the posterior chamber structures is paramount. With this in mind, an UBM examination is highly recommended in any patient where this diagnosis is being considered.

Interestingly, our case series suggests that addressing the pathophysiological process of pigment release (in these cases, by removing the iris chafing mechanism) led to a quick normalization of the IOP. Unlike the primary condition, where the TM is known to be permanently obliterated by a lifelong pigment-induced necrosis, our results would imply that the TM obstruction could be at least partially reversible. Several reasons could be behind this, including earlier diagnosis. Indeed, the pronounced IOL-iris contact could lead not only to aggressive dispersion (with the associated clinical signs of iris atrophy) but also to intraocular inflammation. Both of them could lead to early detection and by acting on the cause of dispersion―ultimately to a shortened TM insult.

Nevertheless, not all patients are well-suited for an IOL exchange. The same principles used in refractive surgery for AC-based lens apply in these situations as well.^10^ Glaucoma presents as a contraindication for angle-based IOL, thus leaving iris-supported IOLs as the most adequate alternative. However, properly securing an IOL to the iris depends on the extent of its atrophy. Implanting these IOLs in such damaged tissue has been associated with abnormal IOL movement leading to aggravated iris atrophy (usually associated with pupil distortion) and corneal endothelial damage.^11-13^ Patient number 3, e.g. already had an endothelial corneal count below the safety threshold for implanting an anterior chamber IOL. Performing intraocular surgery in these conditions―even if considering placing the iris-claw IOL in the posterior chamber―should be done carefully. Breaking the cycle of pigment dispersion and IOP increase should be weighted along with the risks of aggravating corneal damage and decreasing visual acuity. A proper discussion with the patient should be done, explaining potential risks and benefits, to allow an informed decision.

Ultimately, close vigilance should be taken in patients who had a complicated cataract surgery with an IOL placed in the sulcus, as a timely intervention could prevent further clinically-relevant TM damage. This is particularly important as the surgical options in these situations may become narrower overtime.

In summary, this case series illustrates the risk of iatrogenic pigment dispersion glaucoma after improper sulcus IOL implantation, and exemplifies that this condition is potentially treatable by 1OL exchange.
